# SVIP alleviates CCl_4_-induced liver fibrosis via activating autophagy and protecting hepatocytes

**DOI:** 10.1038/s41419-019-1311-0

**Published:** 2019-01-25

**Authors:** Dan Jia, Yuan Yuan Wang, Pin Wang, Yao Huang, David Yuke Liang, Dongmei Wang, Chuandong Cheng, Caihua Zhang, Lianying Guo, Pin Liang, Yang Wang, Yujie Jia, Cong Li

**Affiliations:** 1 Department of Pathophysiology, College of Basic Medical Sciences, Dalian Medical University, Dalian, China; 2 Administration Department, Dalian Medical University, Dalian, China; 3 Faculty of Pharmaceutical Sciences, University of British Columbia, Vancouver, Canada; 4 Department of Experimental Functionality, College of Basic Medical Sciences, Dalian Medical University, Dalian, China; 5 Department of Neurosurgery, The First Affiliated Hospital of University of Science and Technology of China, Anhui Provincial Hospital, Hefei, China; 6 The First Affiliated Hospital of Dalian Medical University, Dalian, China

## Abstract

Prolonged parenchymal cell death leads to activation of fibrogenic cells and extracellular matrix accumulation and eventually liver fibrosis. Autophagy, a major catabolic process of intracellular degradation and recycling, participates in hepatic fibrosis. However, the precise role of autophagy in the pathogenesis of hepatic fibrosis is controversial. The present study aims to investigate the key role of small VCP/p97 interacting protein (SVIP) against CCl_4_-induced hepatic fibrosis via activating autophagy. Autophagy could be activated by SVIP in HepG2 cells, but starvation cannot increase SVIP expression in vitro and in vivo. Moreover, SVIP expression, in agreement with autophagic activity and the volume of lipid droplets, first increases and then decreases during the progression of liver fibrosis with CCl_4_ treatment in vivo and in vivo. Further, overexpression of SVIP can protect HepG2 cells from the toxicity of CCl_4_, which could be enhanced by starvation. Finally, starvation keeps SVIP and autophagy at such high levels in the rat livers that markedly delays the progress of hepatic fibrosis. Probably, the protective effect of SVIP is associated with stabilizing nuclear factor (erythroid-derived 2)-related factor 2 (Nrf2) and transcription factor EB (TFEB). The current study provides insight into the biological role of SVIP and autophagy in regulating hepatic fibrosis, targeting SVIP might be a novel therapeutic strategy in the future.

## Introduction

Liver fibrosis is a common pathological state, in which hepatic stellate cells (HSCs) are activated and then extracellular matrix (ECM) proteins accumulate, usually associated with chronic liver diseases caused by infection, drugs, metabolic disorders, or autoimmune imbalances^1–4^. Liver fibrosis, if not well controlled, will lead to irreversible cirrhosis, even hepatocellular carcinoma^5,6^. So far there has been no efficient clinical therapies to suppress the pathological progression of liver fibrosis except the removal of underlying etiology or liver transplantation^7^. Beyond that, researchers have paid too much attention to inhibit HSCs’ activation rather than to protect the function of the liver. After all, sustained liver parenchymal cells death is essential to initiate the scarring. Therefore, studying the molecular basis of liver fibrosis and developing a new therapeutic approach to protect parenchymal cells and reverse liver fibrosis are needed.

Autophagy is a critical intracellular pathway, that broken organelles and damaged proteins are degraded to provide energy for cellular homeostasis in eukaryotic cell^8–11^. The autophagic pathway proceeds through several phases involving a set of evolutionarily conserved gene products. Upon induction (nutrient deprivation or starvation), inhibition of mTOR complex 1 (mTORC1) activates ULK1/2-Atg13-Atg101-FIP200 complex (Atgs, autophagy-related genes), which initiates an isolation membrane or phagophore formation. Initiation of autophagy is also accompanied by activation of Vps34–Vps15(p150)–Beclin1 complex. Stimulation of Beclin1 complex generates phosphatidylinositol-3-phosphate (PI3P), which promotes autophagosomal membrane nucleation. Autophagosomal elongation requires the Atg5–Atg12 and the microtubule-associated protein light chain 3 (LC3/Atg8) conjugation systems. In addition, LC3 is involved in selective transport of such proteins as p62/SQSTM1 and NBR1 which contain a special LC3-interacting region (LIR) motif serving as adaptors for cargo sequestration, such as mitochondria, protein aggregates, and other cellular structures. GTPase Rab7 is required to complete the stage of autophagosome fusion with lysosome. In the final stage, autophagosomal contents are degraded by lysosomal acid hydrolases and the contents of the autolysosome are released for metabolic recycling^12,13^.

Autophagy, playing a crucial role in regulating adipogenesis, is related to steatosis and liver fibrosis^14^. It could reduce lipid droplets via lipophagy. Otherwise, Long-term lipid load may change membrane lipid composition and decrease the fusion of autophagosome and lysosome both in vitro and in vivo^15^. Thus, in the liver inhibition of autophagy by excessive lipid may lead to lipid droplets accumulation in the hepatocytes (hepatic steatosis)^16^. However, it is reported that activated autophagy served energy for activation and proliferation of HSCs by degrading lipid droplets^5,17,18^. Autophagy inhibits fibrosis by degrading collagen. Activation of autophagy degrades type I collagen in murine liver^19^, reduces oxidative stress and ER stress, and inhibits inflammation to inhibit fibrosis^20^. It also protects hepatocytes from apoptosis^21^. So, the roles of autophagy in HSCs activation and in the progression from steatosis to fibrosis are controversial.

Small p97/VCP-interacting protein (SVIP) localizes to the ER membrane through myristoylation. Originally, SVIP was related to endoplasmic reticulum (ER)-associated degradation (ERAD)^22–25^. SVIP is highly expressed in central nervous system, while it is rarely detected in other organs and tissues. Moreover, SVIP is localized to the ER, cytosol, Golgi apparatus, and very low-density lipoprotein (VLDL) transport vesicles (VTVs) in primary hepatocytes and related to the transportation and secretion of VLDL from ER to Golgi apparatus^26^. Our previous work indicated that SVIP can regulate autophagy. Overexpression of SVIP enhances LC3 lipidation, as well as increases the levels of p62 protein to promote sequestration of polyubiquitinated proteins in starvation-activated autophagy^27^.

Subcutaneous administration of carbon tetrachloride (CCl_4_) has been successfully applied in animal model to study the pathogenesis of liver fibrosis. The liver-specific toxicity induced by CCl_4_ involves the generation of free radicals and subsequent lipid peroxidation^28^, cell membrane damage, impaired mitochondrial function, inflammation, and lipid accumulation in hepatocytes^29^. Thereafter, the histopathologic feature of CCl_4_-induced early stage hepatic fibrosis is enlarged lipid droplets in the cytosol (steatosis)^30^. The present study aimed to investigate the relationship between increased SVIP and activated autophagy during the dynamic process from steatosis to fibrosis and the role of SVIP in protecting parenchymal cell and suppressing liver fibrosis.

## Results

### SVIP activates autophagy in HepG2 cells

**Fig. 1:**
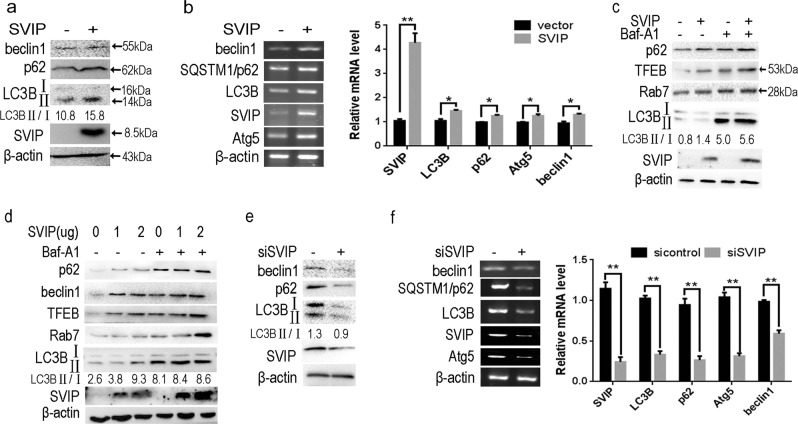
SVIP regulates autophagy in HepG2 cells. **a** Western blots (WB) and **b** PCR showing the expression of Beclin1, p62, LC3B, Atg5 in HepG2 cells transfected with SVIP expressing or control plasmid. **c** and **d** Western blots showing the expression of TFEB, Rab7, and conversion of LC3BII to LC3BI (**c**) in cells transfected with SVIP expressing or/and control plasmid in different dose (**d**). HepG2 cells were transfected with 2 μg total DNA, containing SVIP-expressing plasmid or vector control or 1 μg SVIP-expressing plasmid + 1 μg vector control. Baf-A1, (100 ng/ml) Bafilomycin A1. **e** Western blots and **f** PCR showing the expression of Beclin1, p62, LC3B, Atg5 in silence SVIP HepG2 cells. 24 and 72 h after the plasmid and siRNA transfection, cells were subjected to WB and reverse-transcriptional PCR, respectively. Bar graph indicates the bands’ intensity of mRNA calibrated with β-actin RNA level. LC3-II/LC3-I ratio represents the autophagy flux. Data are presented as mean ± standard division (SD) in three independent experiments. *p < 0.05; **p < 0.01

Previous research showed that SVIP was highly expressed in the central nervous system but not in liver. Moreover, SVIP-activated autophagy in several cell lines by increasing the conversion of LC3-I to LC3-II and expression of p62/SQSTM1^27^. To investigate whether SVIP regulates autophagy in HepG2 cells, we detected the protein and mRNA expression of a series of autophagy-related genes (Atgs) and p62/SQSTM1 that can reflect autophagic activity. Results indicated that the expression of Atgs, including LC3 (mammalian homolog of yeast Atg8), Beclin1 (yeast Atg6), Atg5, and p62/SQSTM1, showed positive association with SVIP over-expression on both protein and mRNA level (Fig. 1a, b); vice versa, their expression reduced in SVIP knockdown cells compared with control cells (Fig. 1e, f). To evaluate the autophagic flux, the ratio between LC3-II and LC3-I was calculated with the help of Bafilomycin A1 which neutralizes the lysosomal pH and blocks autophagosome–lysosome fusion (Fig. 1c, d). Surprisingly, the expression of transcription factor EB (TFEB), a master regulator of lysosomal biogenesis promoting the transcription of autophagy-related genes and p62, increased in a dose-dependent manner as a result of SVIP overexpression (Fig. 1c, d), that explained SVIP increasing the expression of Atgs and p62 on transcriptional level (Fig. 1b, f). All the evidences above suggested SVIP facilitated autophagy in HepG2 cells.

### Starvation-induced autophagy cannot increase SVIP expression

**Fig. 2:**
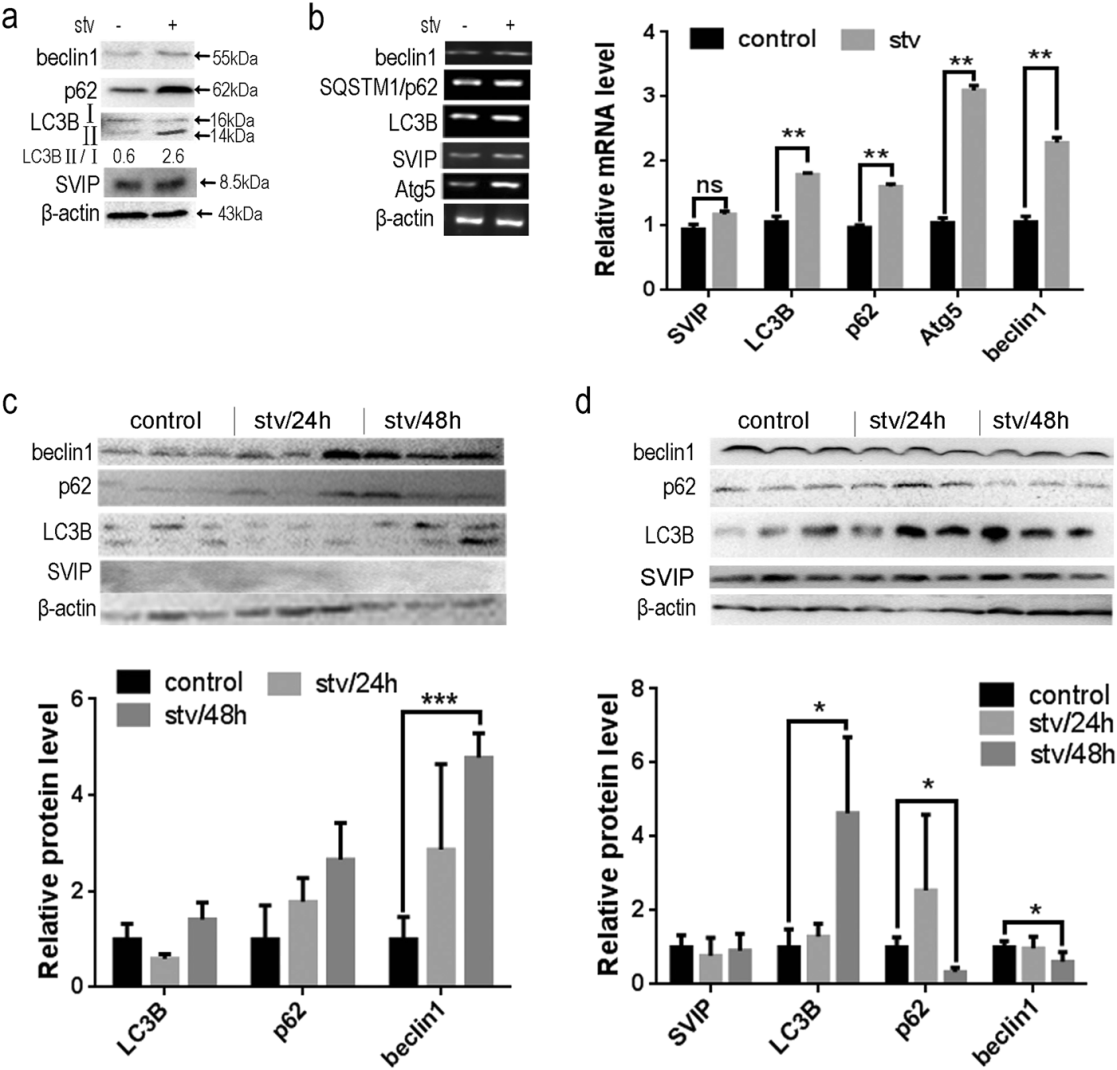
Starvation enhanced autophagy but had no effect on SVIP expression in HepG2 cells and in mice. **a** Western blots (WB) and **b** PCR showing the expression of SIVP as well as Beclin1, p62, LC3B, Atg5. HepG2 cells were maintained in HBSS for 2 h for starvation. Data are presented as mean ± SD in three independent experiments. LC3-II/LC3-I ratio represents the autophagy flux. **c** and **d** Western blots (WB) and PCR showing the expression of SIVP, as well as Beclin1, p62, and LC3B in mouse liver (**c**) or brain (**d**). Mice were starved for an indicated period (*n* = 3). Bar graph indicates the band’ intensity calibrated with β-actin. *p < 0.05; **p < 0.01

Starvation could activate autophagy in vitro and in vivo^31,32^. Moreover, SVIP enhances starvation-induced autophagy^27^. But, it is still unknown whether or not starvation would increase SVIP expression in liver or HepG2 cells. Although LC3, p62, Beclin1, and Atg5 expression changed markedly according to nutritive deprivation, SVIP expression was neither sensitive to starvation in HepG2 cells (Fig. 2a, b) nor in liver (Fig. 2c). Notably, SVIP was still undetectable after 24 or 48 h fasting treatment in liver tissue (Fig. 2c). Similarly, SVIP expression was not influenced by starvation in mouse brain (Fig. 2d).

### SVIP expression is closely associated with autophagic activity during the process of CCl_4_-induced rat liver fibrosis

**Fig. 3:**
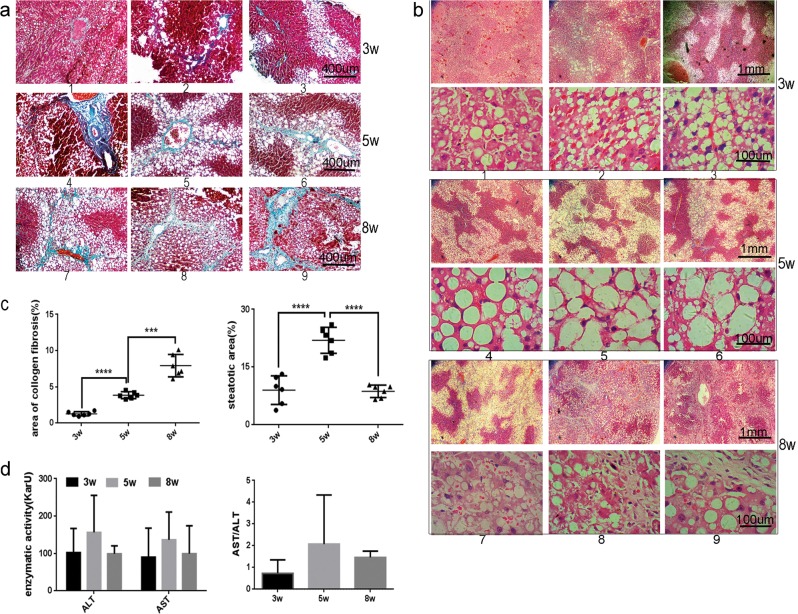

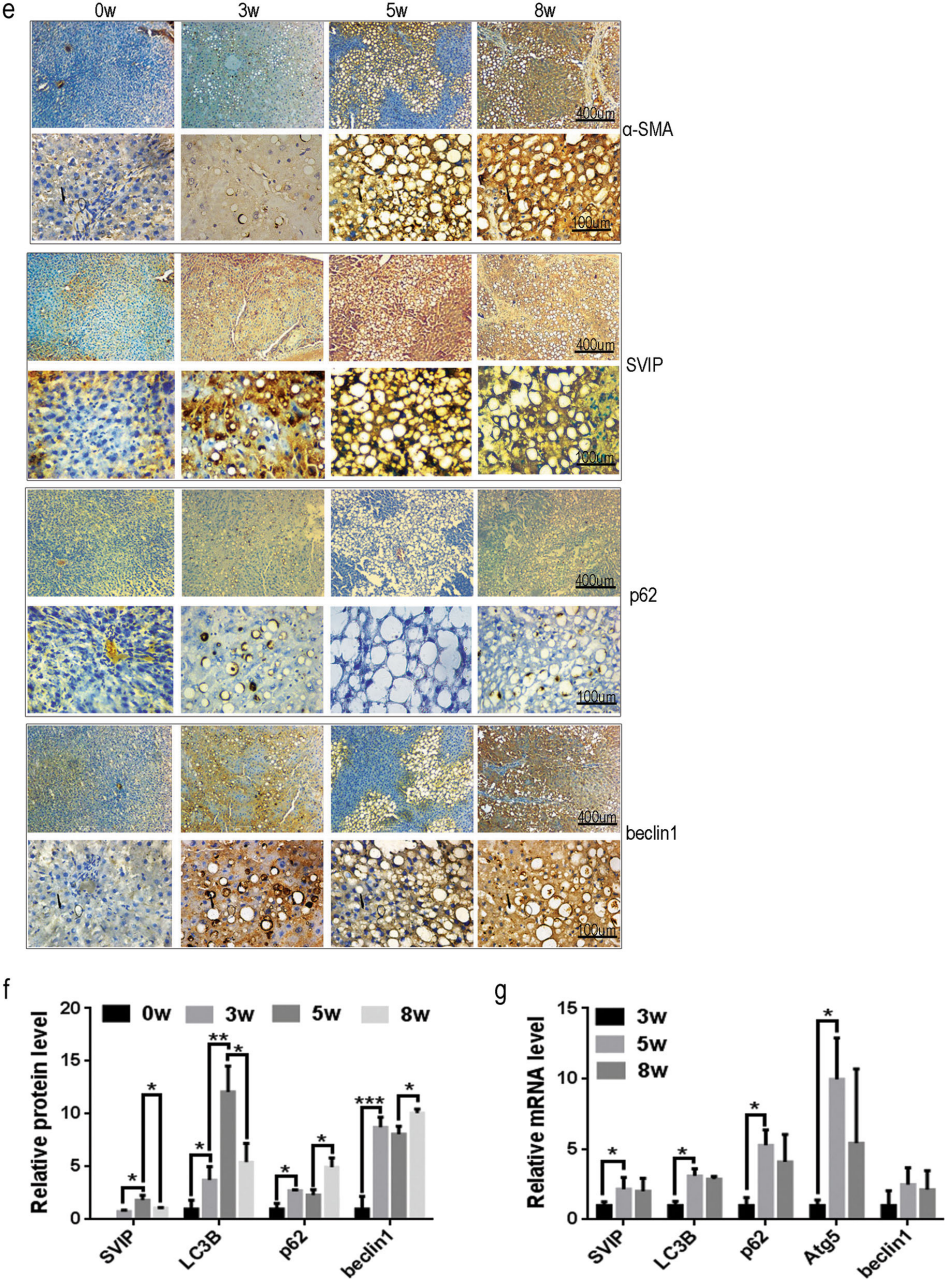
Effect of CCl_4_ treatment on SVIP expression, autophagy and histological alterations. The samples of olive oil (*n* = 3) or CCl_4_ injected Sprague–Dawley (SD) rats for 3, 5, and 8 weeks (*n* = 6) were subjected to the following assays. **a** Masson’s trichrome staining for evaluation of collagen deposition. **b** Representative hematoxylin–eosin (H&E)-stained liver sections from rats exposed to CCl_4_ for 3, 5, or 8 weeks for evaluation of steatosis. **c** The proportions of collagenic (left panel) and steatotic (right panel) areas on Masson’s trichrome-stained and H&E-stained liver sections, respectively. **d** Left panel: representative enzymatic activity of ALT and AST in blood plasma of rats exposed to CCl_4_ for 3, 5, or 8 weeks, right panel: the ratio between AST and ALT. **e** Immunohistochemistry (IHC) analysis of SVIP as well as α-SMA, p62, and Beclin1 expression in liver sections. **f** and **g** Quantitative expressions of SVIP, LC3, p62/SQSTM1, Atg5, and Beclin1 in rat livers were analyzed by WB (**f**) and PCR (**g**). Bar graph indicates the bands’ intensity calibrated with β-actin. Data were presented as mean ± SD, *n* = 3 or 6. *p < 0.05; **p < 0.01, ***p < 0.001

Sprague–Dawley (SD) rats were subjected to CCl_4_ injection for 3, 5, and 8 weeks, respectively. During the progression of hepatic fibrosis, the pathologic features of rats’ livers dramatically changed. Initially, the area of collagen fibers (*A*_ECM_, representing ECM deposition by Masson’s trichrome staining, Fig. 3a) and steatotic area (*A*_S_, H&E staining, Fig. 3b, c) were observed. Fibrotic rat livers showed varying damage: gentle (*A*_S_ < 15%, *A*_ECM_ < 2%), moderate (15% < *A*_S_ < 30%, 2% < *A*_ECM_ < 5%), and severe (*A*_S_ < 15%, *A*_ECM_ > 5%) histological alterations corresponding to 3, 5, and 8 weeks CCl_4_ treatment, respectively (Fig. 3a–c). Interestingly, the dynamics of *A*_S_ displayed a reversed V-shaped curve as well as the size of lipid droplets. *A*_S_ reached the culmination in moderate fibrotic rat livers (Fig. 3a, b, c, e). Furthermore, damage of liver function and severity of liver fibrosis were measured using AST/ALT biochemical analysis and immunohistochemistry (IHC) against α-SMA, a maker for HSCs’ activation^33^, respectively, confirmed the advancing hepatic fibrosis (Fig. 3d, e). Additionally, the expression of related gene products were analyzed using IHC (Fig. 3e), Western blotting assay (WB, Fig. 3f) and reverse transcriptase PCR (Fig. 3g) for evaluating autophagy. The expression of SVIP and LC3 increased at 5 weeks compared with that at 3 weeks, and then decreased at 8 weeks (Fig. 3e, f). This reversed V-shaped curve is consistent with the dynamic change of *A*_S_ in Fig. 3c, that is not merely a coincidence but suggesting *A*_S_ goes along with autophagy/lipophagy. Accumulation of p62 and Beclin1 could be explained as autophagic inhibition in severe fibrotic liver (8 weeks vs. 5 weeks, Fig. 3e, f). Finally, mRNA level of SVIP and Atgs also showed the reversed V-shaped curve from 3rd to 8th week (Fig. 3g). Taken together, during CCl_4_ treatment autophagic activity reached the pinnacle at 5 weeks and then declined at 8 weeks. These data indicated that the expression of SVIP showed a close association with autophagic activity, as well as the size of lipid droplets in fibrotic rat liver.

### Autophagy and SVIP expression in HepG2 cells were first upregulated and then downregulated by CCl_4_ treatment

**Fig. 4:**
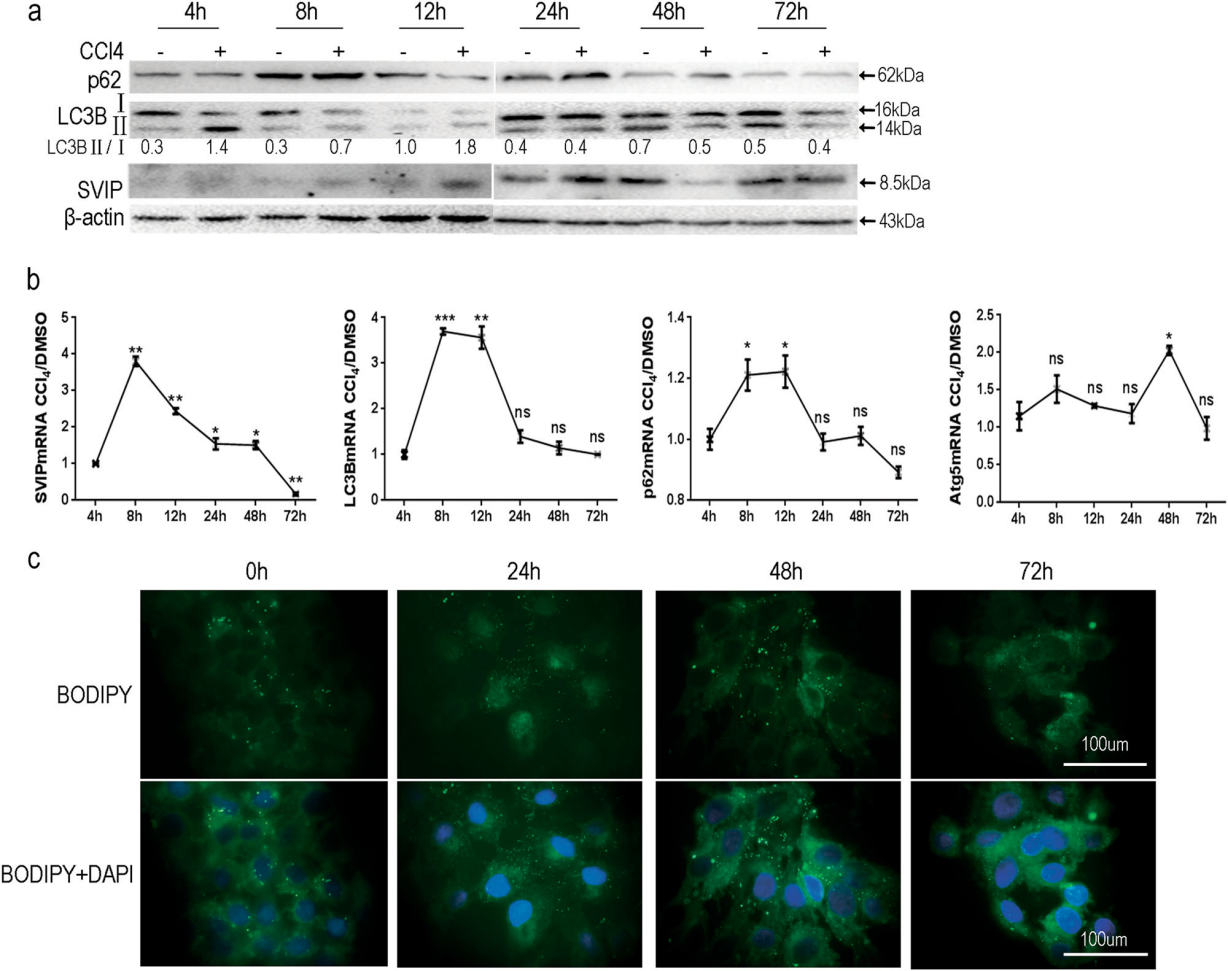
Dynamics of autophagy and SVIP expression in HepG2 cells treated with CCl_4_ or DMSO for an indicated period. **a** Protein expression of p62, LC3B, and SVIP changed according to the period of CCl_4_ treatment in HepG2 cells was detected by WB. **b** The mRNA ratios of CCl_4_-treated to vehicle-treated cells of each gene in HepG2 cells, analyzed using reverse transcription-PCR. Data are presented as mean ± SD in three independent experiments. *p < 0.05; **p < 0.01, ***p < 0.001. **c** Size and volume of lipid droplets in HepG2 cells were constantly changing, stained with BODIPY493/503. Cell nucleus was dyed by DAPI

To investigate whether SVIP or autophagy was regulated by CCl_4_ in vitro, expression of SVIP, Atgs, and p62 were measured in HepG2 treated with CCl_4_. Compared with vehicle treatment, SVIP protein levels increased until 48 h treatment. On the other hand, not only LC3 was obviously down-regulated in CCl_4_-treated HepG2 cells for longer period (48 and 72 h, Fig. 4a), but also inactivation of TFEB and Rab7 by long-period-CCl_4_ treatment (Supplementary Fig. 2, 72 vs. 12 h) and reduced autophagic flux were observed (Supplementary Fig. 1). Also, the transcription levels of these genes were induced during 8–12 h and inhibited after 72 h by CCl_4_, significantly (Fig. 4b). Meanwhile, the result also illuminated the dynamics of lipophagy in a time-dependent manner. From 0 to 48 h, the size and quantity of lipid droplets were increasing with CCl_4_ treatment, but both decreased during 48–72 h (Fig. 4c). In consistent with the result in vivo, CCl_4_ induced the expression of SVIP and autophagy in short time, but inhibited them in long period in vitro.

### Starvation enhanced SVIP-mediated protection of HepG2 cells against CCl_4_ toxicity

**Fig. 5:**
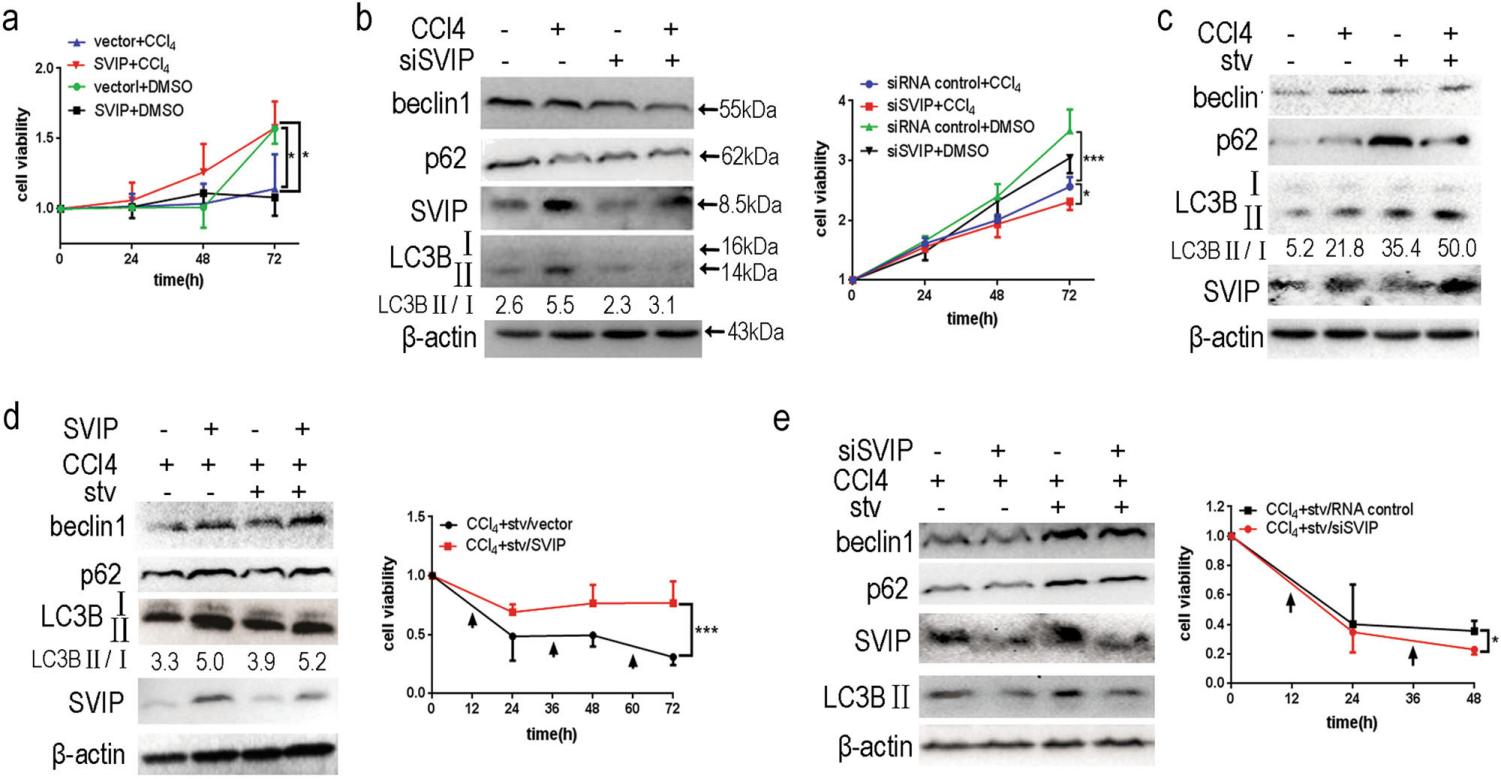
Effect of SVIP and starvation on cell viability and autophagy in CCl_4_-treated HepG2 cells. **a** MTT assay showing the viability of HepG2 cells transfected with SVIP-expressing plasmid or vector control treated with CCl_4_ or DMSO for 0–72 h. **b** Western blots (left panel) showing the expression of SVIP, Beclin1, p62, and LC3B in HepG2 cells transfected with siSVIP or siRNA control treated with CCl_4_ or DMSO for 24 h. MTT assays (right panel) showing the viability of HepG2 cells transfected with siSVIP or siRNA control treated with CCl_4_ or DMSO for 0–72 h. **c** Western blots showing the expression of SVIP, Beclin1, p62, and LC3B in HepG2 cells treated with CCl_4_ or DMSO (12 h) in the absence and presence of starvation (2 h). **d** Western blots (left panel) and MTT assays (right panel) showing SVIP, p62, Beclin1, and LC3B expression and cell viability of CCl_4_-treated HepG2 cells transfected with SVIP expressing plasmid or vector in the presence or absence of starvation. Starvation started at 12, 36, and 60 h as indicated by arrows. **e** Western blots (left panel) and MTT assays (right panel) showing the expression of SVIP, Beclin1, p62, and LC3B in CCl_4_-treated HepG2 cells transfected with siSVIP or siRNA control in the absence and presence of starvation. 72 h after siRNA transfection, cells were subjected to CCl_4_ treatment in the absence and presence of starvation (45 min). Starvation started at 12 and 36 h as indicated by arrows. Data are presented as mean ± SD in three independent experiments. LC3-II/LC3-I ratio represents the autophagy flux. *p < 0.05; **p < 0.01; ***p < 0.001

To clarify the biological functions of SVIP and autophagy on CCl_4_-treated cells, we tested the cell viability with SVIP overexpression or SVIP depletion followed by CCl_4_ treatment in the attendance or absence of nutrient depletion. In consistent with our previous finding, SVIP overexpression led to apoptosis in glioblastoma cell line U87MG^34^, it delayed the growth of HepG2 cells (Fig. 5a). However, in the attendance of CCl_4_, SVIP overexpression surprisingly eliminated CCl_4_-induced cellular toxicity (Fig. 5a). Meanwhile, SVIP depletion by siRNA not only led to autophagy inhibition, but also increased the sensitivity of HepG2 cells to CCl_4_ (Fig. 5b). Next, we investigated whether starvation, as an inducer of autophagy, could relieve of CCl_4_ toxicity and improve the survival of HepG2. Strikingly, starvation, although cannot induce SVIP expression alone, increased SVIP expression following with CCl_4_ treatment and activated autophagy in HepG2 cells (Fig. 5c). Furthermore, starvation enhanced autophagy and cell viability in CCl_4_-treated HepG2 cells overexpressing SVIP (Fig. 5d). Finally, with CCl_4_ treatment SVIP knockdown by siRNA significantly inhibited autophagy and led to cell death even under a condition of autophagic activation by starvation (Fig. 5e). Taken together, SVIP is upregulated by starvation in CCl_4_-treated HepG2 cells and alleviates the harm of CCl_4_ to HepG2 cells.

### Fasting alleviates CCl_4_-induced rat liver fibrosis via enhancing autophagy and SVIP expression

**Fig. 6:**
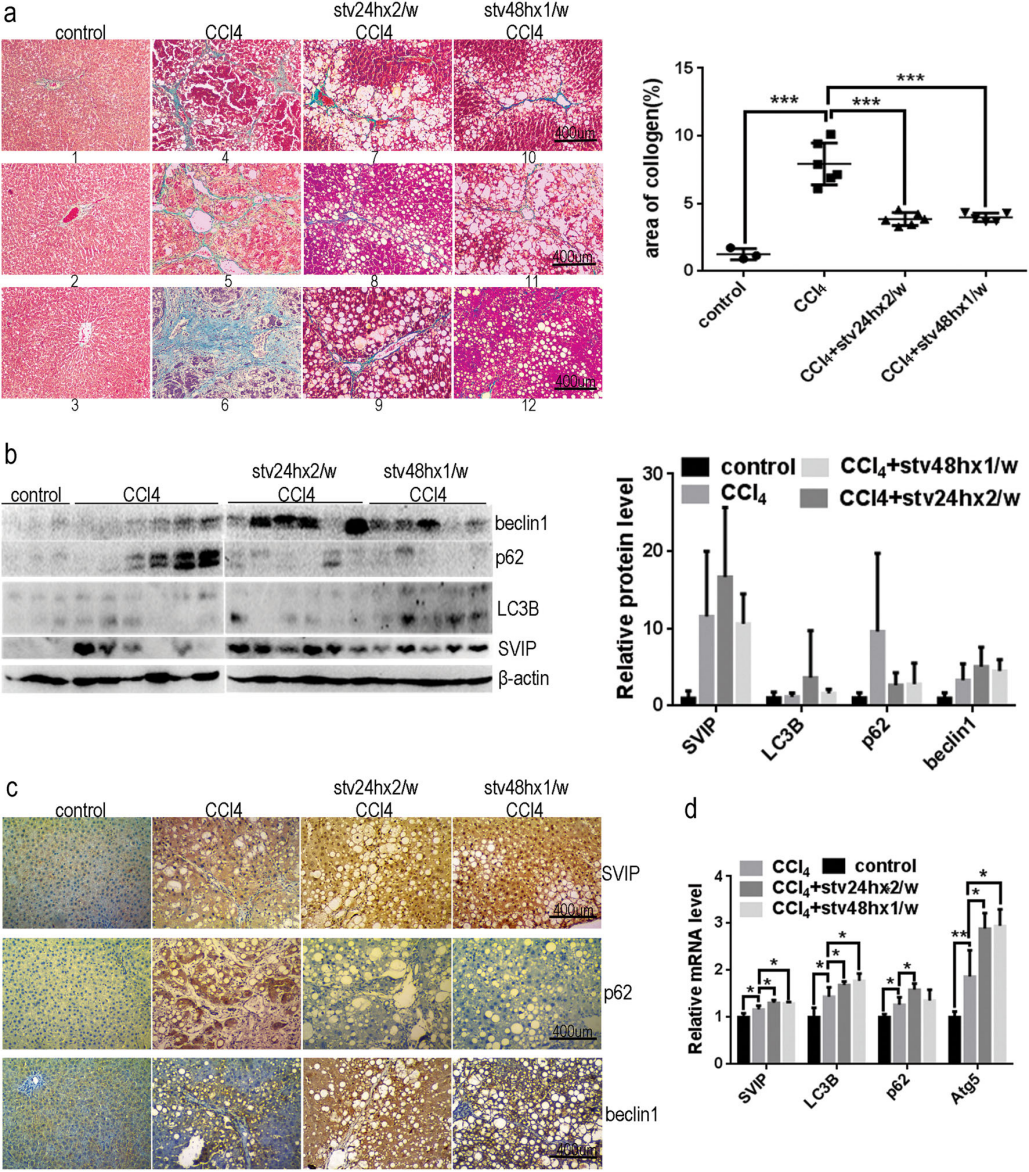
Starvation increasing SVIP expression alleviated CCl_4_-induced liver fibrosis. Twenty SD rats were injected subcutaneously with 1 ml/kg 50% (v/v) CCl_4_ or olive oil (control, *n* = 3, 0 death) twice a week for 8 weeks, CCl_4_-treated rats were starved for 24 h twice weekly (stv24hx2/w CCl4, *n* = 6, 1 death) or 48 h once weekly (stv48hx1/w CCl_4_, *n* = 5, 0 death) or without starvation (CCl_4_, *n* = 6, 2 death), the liver tissues were subjected to the following assays. **a** Masson’s trichrome staining of liver sections from rats exposed to CCl_4_ or olive oil (left panel) and the proportions of collagenic areas on Masson’s trichrome-stained sections (right panel). **b** Western blots showing protein expression of SVIP, Beclin1, p62, and LC3 in liver tissues (left panel) and quantification of relative protein levels (right panel) calibrated with β-actin. **c** Immunohistochemistry (IHC) analysis of SVIP, p62, and Beclin1 expression in liver sections. **d** PCR analysis of mRNA levels of SVIP, p62, LC3B, and Atg5. Relative mRNA levels were calibrated with β-actin. Data are presented as mean ± SD, *p < 0.05; **p < 0.01; ***p < 0.001

The protective effect of nutrient deprivation on hepatocytes was investigated in vivo. Rats were subject to 8-week-CCl_4_ injection subcutaneously with or without short period starvation, including 24-h fasting twice per week or 48-h fasting once per week. After 8 weeks treatment, the rats subjected to 48 h starvation once per week, but not to 24 h starvation twice per week, showed reduced body weight (Supplementary Fig. 3). First, the area of collagen fibers and damage of liver were measured. The result showed a remission of hepatic fibrosis in repeated fasting groups characterized by reduced area of collagen fibers compared with CCl_4_ group (Fig. 6a). However, as the signs of liver function, ALT and AST values were not improved by the fast, because the basal level of ALT and AST might be elevated by starvation (Supplementary Fig. 4), so ALT or AST is not suitable for the characterization of liver function in case of starvation. Next, to reveal whether SVIP involved in attenuated rat liver fibrosis, we investigated the expression of SVIP and autophagy-related proteins in these four groups (Fig. 6b). The result indicated that p62 accumulation was a sign of autophagy inhibition in CCl_4_ group compared with control (Fig. 6b, c), because in those rat livers lower SVIP expression always accompanied with higher level of p62 (Fig. 6b). In addition, SVIP and autophagy were induced in fasting rats, especially in 24 h twice per week starvation group, compared with CCl_4_ group (Fig. 6b, c, d). The levels of mRNA of autophagy-related proteins and SVIP were increased in fasting rats (Fig. 6d). Our results suggested that starvation increases the expression of SVIP together with activation of autophagy to alleviate CCl_4_-induced liver fibrosis in vivo.

## Discussion

**Fig. 7:**
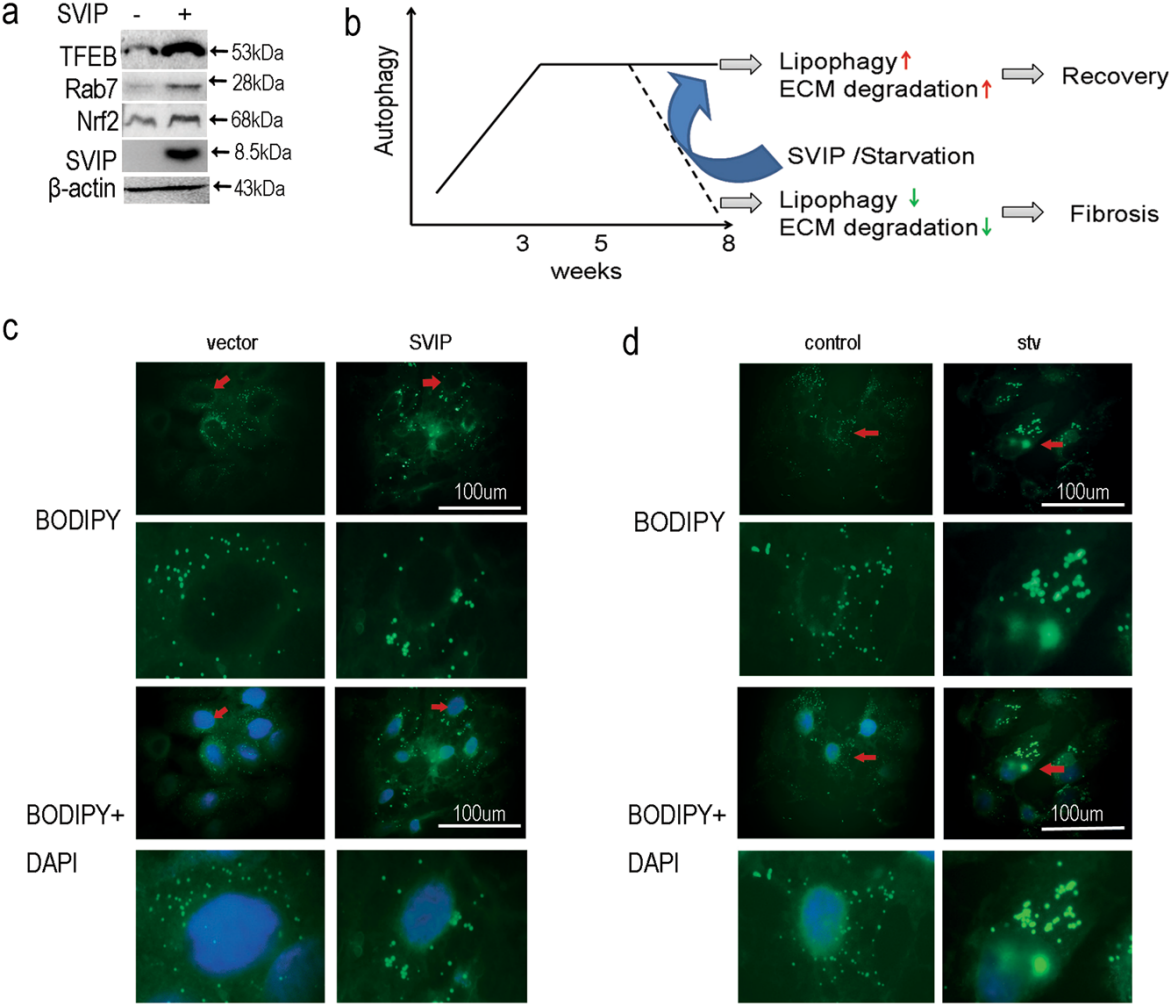
SVIP and starvation, promoting autophagy/lipophagy and converging lipid droplets, protect hepatocytes. **a** Western blots showing the expression of Nrf2, TFEB, and Rab7 in HepG2 cells transfected with SVIP expressing plasmid or vector control. **b** Schematic view of SVIP and starvation’s effects on autophagy/lipophagy determining the prognosis of the steatotic liver based on a CCl_4_-induced fibrosis model. During 5 and 8 weeks, enhanced SVIP expression and autophagy would increase lipophagy and extracellular matrix (ECM) degradation to reverse liver fibrosis. **c** and **d** Representative fluorescence images of the quantity and size of lipid droplets in HepG2 cells transfected with SVIP expressing plasmid or vector control (**c**) and in the absence and presence of starvation (stv, 2 h) (**d**). Lipid droplets and nuclei were stained with BODIPY493/503 (green) and DAPI (blue), respectively. Scale bar, 100 μm. Red arrows indicated the magnified areas in lower panels

SVIP not only regulates ERAD by interacting p97/VCP competitively with gp78, but also activate autophagy in several cell lines^22–25,27^. In the present study, SVIP activated autophagy in HepG2 cells. More than that, SVIP and autophagy were initially activated and finally suppressed simultaneously in the progression of CCl_4_-induced hepatocytes’ damage in vitro and in vivo. Starvation did not increase the expression of SVIP. However, in the attendance of CCl_4_, starvation induced autophagy and SVIP expression. Both SVIP and starvation showed protecting effect against CCl_4_ toxicity (Fig. 7b). Autophagy plays an important role in degrading collagen (ECM) and reducing apoptosis, oxidative stress, ER stress, and inflammation^20,21^. Whereas SVIP alone can act as an anti-damage factor (Fig. 5a). Therefore, recent results prompted us to investigate the exact mechanism of SVIP in autophagy to inhibit liver fibrosis.

The mechanism by which SVIP protects hepatocytes may include reducing the accumulation of fatty acid and enhancing the antioxidation in the steatosis liver. Initially, studies have shown that hepatocytes suffered from CCl_4_ because of the accumulation of fatty acid and lipid peroxidation caused cytotoxicity^28,29,35^. Therefore, SVIP could regulate autophagy/lipophagy reducing the accumulation of lipid. Moreover, SVIP has a VCP-interacting motif (VIM), while ubiquitin ligases Hrd1 and STUB1/CHIP rely on their VCP-binding motifs (VBMs) to degrade the substrates. Both VIM and VBM can bind to ND1 domain of VCP and the affinity of ND1 to VIM is higher than that to VBM^25^. So increased SVIP competitively inhibits Hrd1 and STUB1/CHIP binding to VCP and reduces the degradation of their substrates. Nuclear factor (erythroid-derived 2)-related factor 2 (Nrf2) and TFEB, the substrates of Hrd1 and STUB1/CHIP, are transcription factors regulating the genes associated with antioxidant stress and lysosomal biogenesis^36–38^, respectively. Interestingly, both Nrf2 and TFEB were up-regulated by SVIP over-expression (Fig. 7a). Thus, SVIP may be able to regulate autophagy (via regulating TFEB) and to protect hepatocytes (via regulating Nrf2). SVIP overexpression also increased Rab7 dramatically (Fig. 7a). Concerning that Rab7 plays a central role in autophagosome–lysosome fusion and lysosme–lipid droplet fusion^39^ and that Rab7 may be the downstream protein of TFEB^40^, SVIP could be a regulator of hepatocellular lipid droplet catabolism. Last but not least, it was reported that increased expression of SVIP contributed to the secretion of VLDL to reduce the hepatocytotoxicity of fatty acids^26^.

The expression of SVIP was sensitive to CCl_4_ in rat livers and HepG2 cells. The mechanism may relate to activation of the ubiquitin-proteasome pathway. Some studies have shown that SVIP was increased and ubiquitin-proteasome pathway was activated by ER stress^36,41^. When CCl_4_ causes ER stress in hepatocytes, ER-associated degradation and the expression of SVIP will increase. Autophagy is activated by SVIP to protect hepatocytes (Fig. 5). If SVIP was depleted by siRNA, autophagy was inhibited (Fig. 5e). Meanwhile, HepG2 cells were more sensitive to CCl_4_ toxicity.

Autophagy is closely related to hepatic steatosis and fibrosis^42^. However, the dynamics of autophagy in the process from steatosis to fibrosis was rarely reported. Our results indicated autophagy was activated along with the development of hepatic steatosis, and then inactivated once progressed to fibrosis (Fig. 7b). Here, in a CCl_4_-treatment model, liver fibrosis could be alleviated by activating autophagy or expression of SVIP (Fig. 7b). Interestingly, in our study the volume of lipid droplets continuously increased accompanied with activated autophagy (Fig. 3a–c and Fig. 4c), while either SVIP overexpression or starvation in HepG2 cells also showed enlarged lipid droplets (Fig. 7c, d). Lipid accumulation (featured by enlarged lipid droplets) was thought to reflect the imbalance between adipogenesis and autophagy/lipophagy and to presage hepatic damage^16^. In our study, over-speed adipogenesis and elevated autophagy occurs simultaneously in steatotic liver but not in severe fibrotic liver (Fig. 3). When it progressed to fibrosis, liver function was affected severely, diminished autophagy and decelerated adipogenesis were shown and the volume of lipid droplets decreased. Taking what is above-mentioned into account, the dynamics of autophagy, steatotic area (Fig. 3b, c), the expression of SVIP, TFEB, and transcription of the downstream genes (Fig. 3f, g, Fig. 4a, b and Supplementary Figs. 1 and 2) including LC3, p62/SQSTM1, Beclin1, Atg5, and Rab7 in vitro or in vivo are all in reverse V-shaped curves during the progression of fibrosis. It is not coincidental that we consider SVIP mediated this unusual tendency by stabilizing TFEB (Fig. 1c, d) and increased downstream genes transcription (Fig. 1b, f).

In conclusion, the expression of SVIP is closely associated with the level of autophagy during the development of CCl_4_-induced hepatic fibrosis. SVIP can induce autophagy and suspend hepatic fibrosis. It is very meaningful to study the molecular mechanism of SVIP activating autophagy, which may provide a new theoretical basis for anti-fibrosis in the future.

## Materials and methods

### Animals

For the starvation model, mice (8 weeks of age) were subjected to a 5 h fast and a 2 h re-feeding for synchronization^43^ (9:00 a.m. to 16:00 p.m.) prior to food deprivation for 0, 24, or 48 h (*n* = 3, started from 16:00 p.m.) and sacrificed immediately. For the liver fibrosis model, 18 SD rats (SPF Center, Dalian Medical University, China) with an initial body weight between 250 and 300 g were injected with 1 ml/kg 50% (v/v) CCl_4_ in olive oil (Guangfu, Tianjin, China) twice a week^29^ for 3, 5, and 8 weeks (*n* = 6), control group (*n* = 3) injected the same dosage of olive oil for 3, 5, and 8 weeks. For the liver fibrosis and starvation model, CCl_4_ or olive oil injection referred to the liver fibrosis model, and CCl_4_-treated SD rats were subjected to fast for 0 (*n* = 6), 24 h (twice per week, *n* = 6), or 48 h (once per week, *n* = 5) following the synchronization step. Liver tissues and serum were collected for analysis. During starvation these animals had free access to drinking water.

All experiments were approved by the Ethical Committee on Animal Experimentation of Dalian Medical University.

### Cell culture, transfection, and treatment

HepG2 cells were purchased from Keygen (Nanjing, China). HepG2 cells were cultured in Dulbecco’s Modified Eagle’s Medium (DMEM) (Invitrogen) supplemented with 10% fetal bovine serum (FBS). HepG2 cells were treated with 0.05% CCl_4_ or vehicle (DMSO)^28^ for an indicated time period. Bafilomycin A1 (100 ng/ml, Selleck, China) was used for 2 h to inhibit lysosomal contents degradation. In the starvation experiments, HepG2 cells were treated with Hanks balanced salt solution (HBSS, Hyclone) for 2 h. HepG2 cells were transfected with Lipofectamine 2000 (Invitrogen, USA) for the transfection of plasmids encoding SVIP, siSVIP (the sequences as described previously^27^) and control siRNA performed with according to the manufacturer’s instructions.

### Histopathological analysis

Formalin-fixed paraffin-embedded liver sections were subjected to hematoxylin and eosin (H&E) staining and immunohistochemistry (IHC) as described previously^36^. Masson’s trichrome staining was performed according to the manufacturer’s instructions using the trichrome stain (Masson) kit (Keygen, Nanjing, China). Microscopy images were acquired using Olympus IX71 microscope and analyzed using Image Pro-plus.

### Western blot analysis

Protein level in liver tissue from rats and mice or cells was determined as described previously^27^. Primary antibodies, polyclonal rabbit anti-LC3B was purchased from CST (USA), anti-p62, and Beclin1 were purchased from Santa Cruz Biotechnology, USA. Immunoreactive bands were visualized by use of ECL and the ChemiDoc XRS Imaging System. Densitometric analysis was performed by Image Lab software (Bio-Rad) and the protein band intensity was calculated by normalizing against the β-actin (Proteintech, China) band intensity. LC3-II/LC3-I ratio was calculated to evaluate LC3 lipidation and autophagy flux.

### Reverse transcriptase PCR

Total RNA was extracted from cell pellets or fresh tissues using the TRIzol reagent (Invitrogen, USA). RT-PCR was undertaken with TransScript First-Strand cDNA Synthesis SuperMix (Transgen Biotech, Beijing, China) and 2 × EasyTaq PCR SuperMix (Transgen Biotech, Beijing, China) according to the manufacturer’s instructions. The primers (5’–3’) include SVIP (forward: TGTGCTTCCCGTGTCCCG; reverse: TGCCTCCACCGACTGGATAT), LC3B (forward: AAAAGGGACGTTACCAGCGG; reverse: GAGGGACTGTTTCCAGGGAC), Beclin1 (forward: GCTCAGTATCAGAGAGAATA; reverse: GTCAGAGACTCCAGATATGA), Atg5 (forward: ATGTGCTTCGAGATGTGTG; reverse: GTGTGCCTTCATATTCAAACC), SQSTM1/p62 (forward: CACGGTGAAGGCCTATCTACTG; reverse: TCACTGGAGAAGGCGACCAA), and β-actin (forward: GCTCGTCGTCGACAACGGCT; reverse: CAAACATGATCTGGGTCATCTTCTCT).

### Biochemical analysis

The amounts of alanine aminotransferase (ALT), aspartate aminotransferase (AST) in serum were estimated according to the manufacturer’s instructions from GPT/ALT kit or GOT/AST kit (Jiancheng, Nanjing, China).

### MTT/cell viability assay

Cell viability was assessed with MTT analysis as described previously^28^. After different treatments (DMSO or 0.05% CCl_4_) and different incubation times (0, 24, 48, 72), absorbance of the wells was read in a scanning well microculture plate reader at test and reference wavelengths of 570 and 630 nm.

### Fluorescence microscopy

HepG2 cells were fixed with 10% formaldehyde for 30 min at 4 °C and dyed with BODIPY493/503 (1:1000) (Tokyo Chemical Industry, Japan) for 15 min. After washing with PBS, nucleus was dyed with DAPI (Thermo, USA) for 20 min. To monitor the autophagic flux, HepG2 cells stably expressing tandem fluorescent protein-tagged human LC3 were created by transfecting pHluorin-mKate2-LC3 plasmid, a gift from Isei Tanida (Addgene plasmid #61458), and selected with puromycin. Lipid droplets and autophagosome/autolysosome were detected with fluorescent microscopy, the images were acquired and analyzed using Image Pro-plus.

### Statistical analysis

All statistical analyses were performed in GraphPad Prism Version 6 (GraphPad Software, San Diego, USA) statistical software. Data are presented as means ± standard division of at least three independent experiments. Data were analyzed by two-tailed Student's *t*-test for comparisons between two groups, or one-way analysis of variance (ANOVA) with post hoc Bonferroni multiple comparison test for comparisons involving greater than two groups. A *P*-value < 0.05 was considered significant.

## Supplementary information

SUPPLEMENTAL FIGURES
