# Supplementary material for: SVIP alleviates CCl4-induced liver fibrosis via activating autophagy and protecting hepatocytes
Source: Cell Death Dis. 2019 Jan 25;10(2):71. doi: 10.1038/s41419-019-1311-0 (PMC6347612; doi:10.1038/s41419-019-1311-0)
Supplement: Supplementary file 1 — SUPPLEMENTAL FIGURES [file 41419_2019_1311_MOESM1_ESM.ppt]

## Slide 1
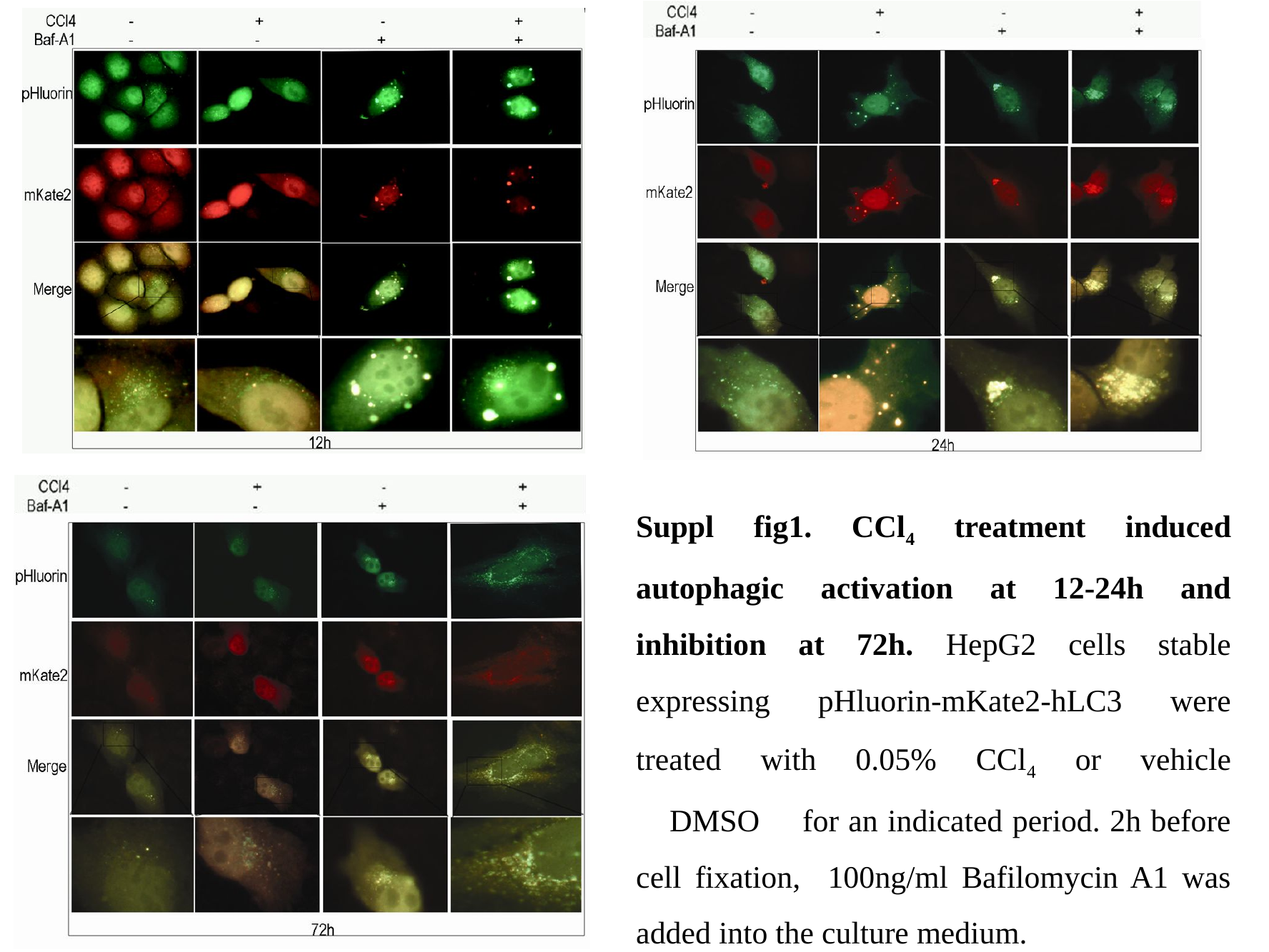

Suppl fig1. CCl4 treatment induced autophagic activation at 12-24h and inhibition at 72h. HepG2 cells stable expressing pHluorin-mKate2-hLC3 were treated with 0.05% CCl4 or vehicle （DMSO）for an indicated period. 2h before cell fixation, 100ng/ml Bafilomycin A1 was added into the culture medium.

## Slide 2
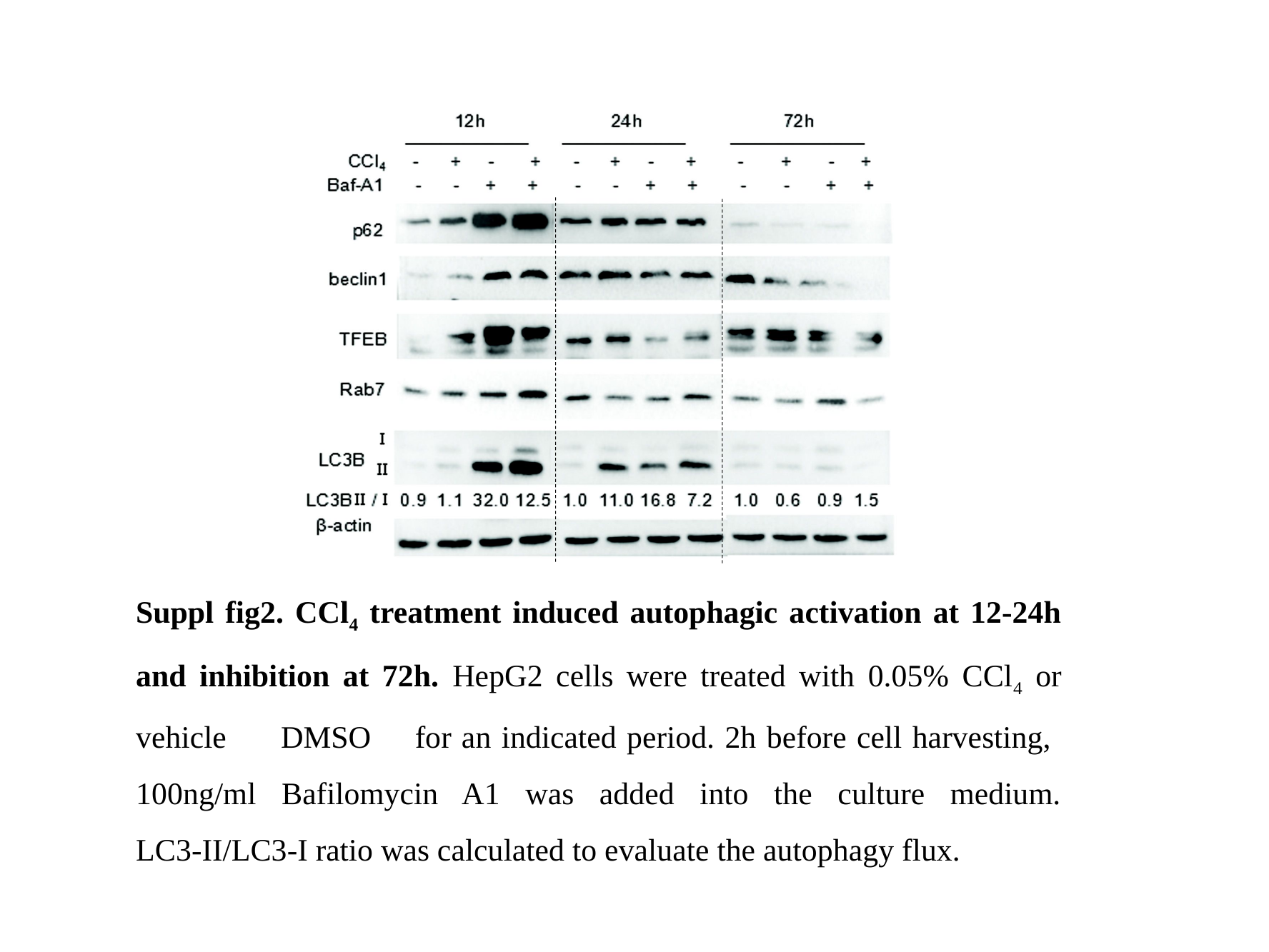

Suppl fig2. CCl4 treatment induced autophagic activation at 12-24h and inhibition at 72h. HepG2 cells were treated with 0.05% CCl4 or vehicle （DMSO）for an indicated period. 2h before cell harvesting, 100ng/ml Bafilomycin A1 was added into the culture medium. LC3-II/LC3-I ratio was calculated to evaluate the autophagy flux.

## Slide 3
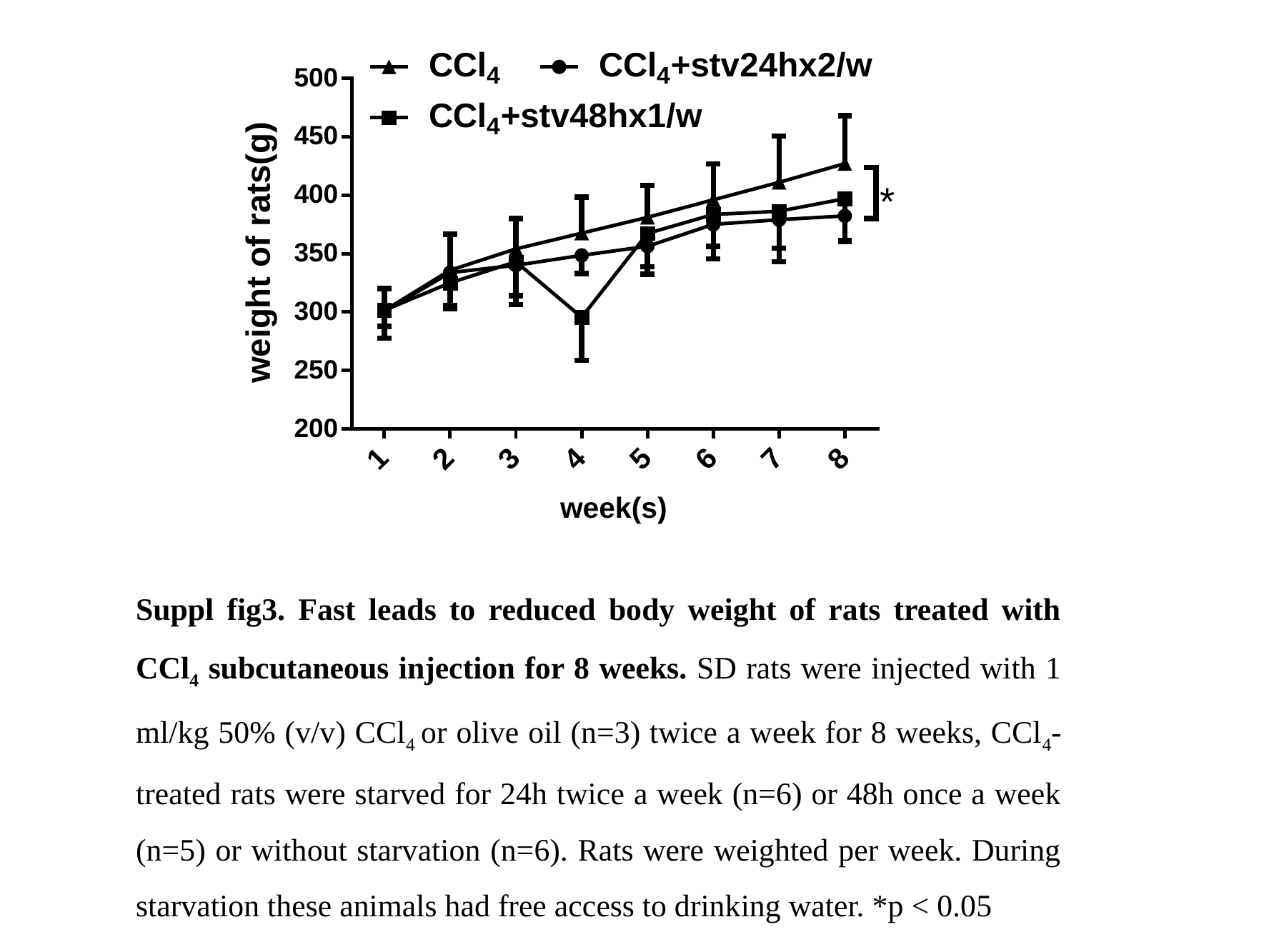

Suppl fig3. Fast leads to reduced body weight of rats treated with CCl4 subcutaneous injection for 8 weeks. SD rats were injected with 1 ml/kg 50% (v/v) CCl4 or olive oil (n=3) twice a week for 8 weeks, CCl4-treated rats were starved for 24h twice a week (n=6) or 48h once a week (n=5) or without starvation (n=6). Rats were weighted per week. During starvation these animals had free access to drinking water. *p < 0.05

## Slide 4
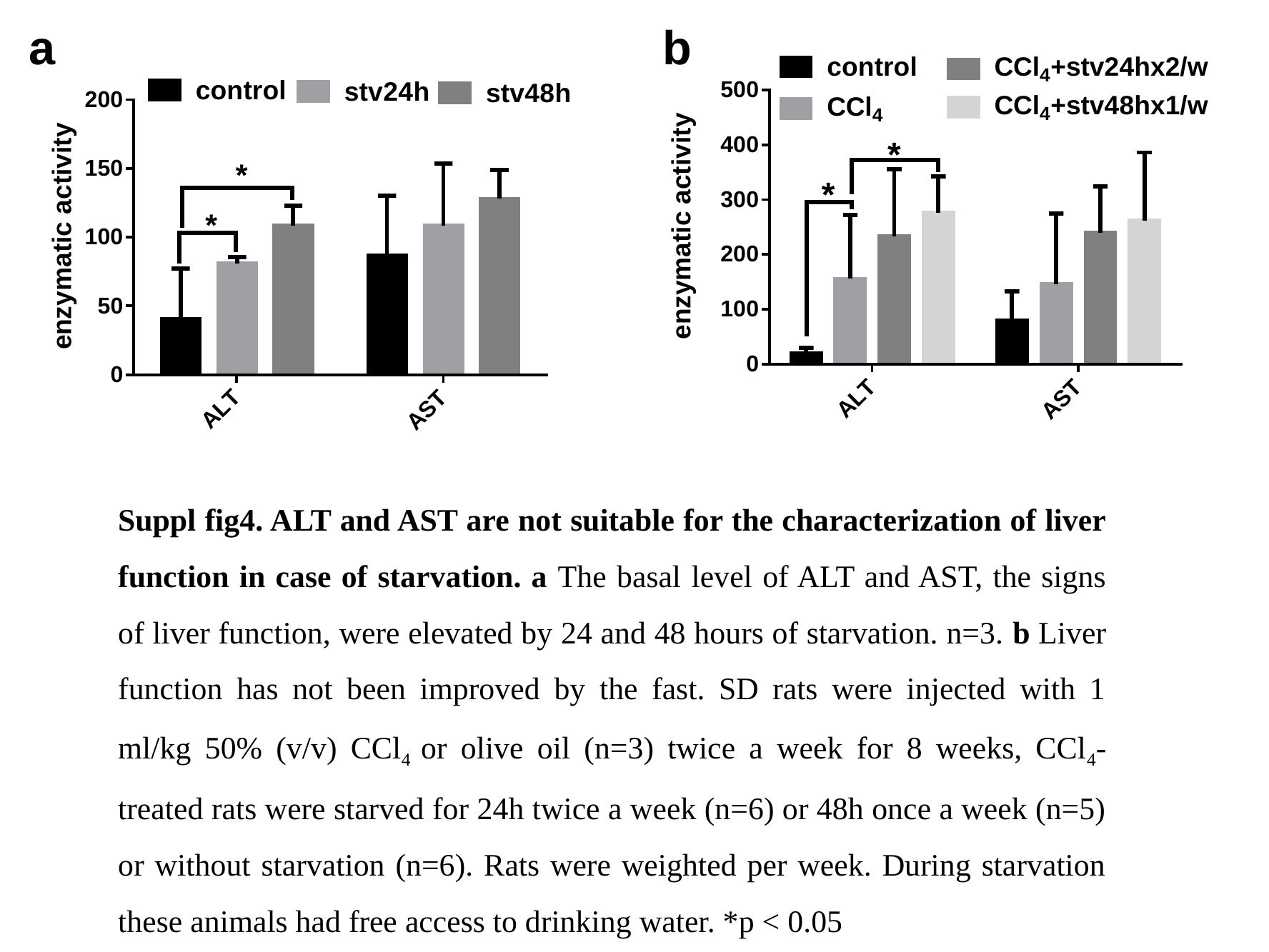

b
a
Suppl fig4. ALT and AST are not suitable for the characterization of liver function in case of starvation. a The basal level of ALT and AST, the signs of liver function, were elevated by 24 and 48 hours of starvation. n=3. b Liver function has not been improved by the fast. SD rats were injected with 1 ml/kg 50% (v/v) CCl4 or olive oil (n=3) twice a week for 8 weeks, CCl4-treated rats were starved for 24h twice a week (n=6) or 48h once a week (n=5) or without starvation (n=6). Rats were weighted per week. During starvation these animals had free access to drinking water. *p < 0.05
